# An interpretable XGBoost model for predicting arteriovenous fistula dysfunction in end stage renal disease

**DOI:** 10.3389/fsurg.2026.1860573

**Published:** 2026-08-17

**Authors:** Run Zhang, Qiongfang Zhang, Xiaolan Zhao, Yu Zhou, Mengjie Cai, Na Yin, Pan Xie, Yi Wu, Lihua Fu

**Affiliations:** Department of Nephrology, The First Hospital Affiliated to Army Medical University, Chongqing, China

**Keywords:** arteriovenous fistula, hemodialysis, inflammatory markers, machine learning, XGBoost

## Abstract

**Background:**

Arteriovenous fistula (AVF) dysfunction remains a major challenge in patients with end-stage renal disease (ESRD) undergoing hemodialysis. This study aimed to develop and evaluate an interpretable machine learning model based on clinical variables and preoperative laboratory parameters to predict AVF dysfunction.

**Methods:**

This retrospective study included patients with ESRD who underwent creation of a new autogenous AVF between January 2021 and December 2023. AVF dysfunction was defined as clinically relevant inadequate access function caused by AVF stenosis, occlusion, or thrombosis within 1 year after AVF creation. Candidate predictors included preoperative clinical variables, routine laboratory parameters, and derived composite indices. The overall cohort was randomly divided into train and test cohorts. Five machine learning models, including XGBoost, random forest, Naive Bayes, support vector machine, and logistic regression, were developed and compared.

**Results:**

Among the 696 patients, 130 (18.7%) developed AVF dysfunction within 1 year. Through recursive feature elimination-based feature selection, six predictors were selected for the final model, including hemoglobin (HB), aggregate index of systemic inflammation (AISI), dialysis vintage, calcium–phosphorus product, triglycerides, and C-reactive protein-to-albumin ratio. The XGBoost model showed favorable discrimination, with AUCs of 0.915 in the train cohort and 0.912 in the test cohort, as well as favorable overall performance in terms of area under the precision–recall curve, sensitivity, specificity, positive and negative predictive values, accuracy, and *F*1-score. The calibration plot, Brier scores, calibration intercepts, and calibration slopes of XGBoost were also acceptable. Decision curve analysis indicated positive net benefit across a range of threshold probabilities. SHAP analysis identified HB, AISI, and dialysis vintage as the leading contributors to model predictions.

**Conclusion:**

An interpretable XGBoost model based on six preoperative variables was developed to predict AVF dysfunction within 1 year after AVF creation in patients with ESRD.

## Introduction

Arteriovenous fistula (AVF) remains the preferred vascular access for hemodialysis because of its lower risk of infection, longer patency, and improved patient survival compared with catheters and grafts (1–3). However, AVF dysfunction, most often resulting from stenosis or thrombosis and leading to inadequate blood flow, is common in clinical practice and is associated with inadequate dialysis, increased hospitalization, and higher mortality. Recent data suggest that a substantial proportion of patients require repeated endovascular or surgical interventions to maintain AVF patency, underscoring the need for early identification of patients at high risk of dysfunction (2).

A variety of demographic, clinical, and anatomical factors, such as age, diabetes, dialysis vintage, and vascular quality, have been linked to AVF failure, and several risk prediction models have been proposed over the past decade (4, 5). Recent machine learning studies have further demonstrated the feasibility of data-driven prediction across a range of AVF-related outcomes, including early AVF failure, AVF maturation or successful clinical use, 1-year successful clinical use, long-term patency, and specific failure mechanisms such as stenosis, thrombosis, and occlusion (6–13). Artificial intelligence approaches based on vascular sound or imaging features have also been explored for automatic detection of AVF stenosis (14). These studies have provided valuable insights into AVF risk prediction and surveillance. Nevertheless, many existing models have focused on heterogeneous endpoints and have used diverse predictor sets, including imaging, acoustic, postoperative surveillance, or access-history variables, which may not always be available before AVF creation. Therefore, a complementary model based on routinely available preoperative variables may help identify patients at increased risk of late AVF dysfunction and support more individualized postoperative access surveillance.

In this context, routine preoperative laboratory parameters are attractive predictors because they are objective, inexpensive, reproducible, and readily available before AVF creation. Moreover, derived inflammatory, nutritional, coagulation-related, and metabolic indices may reflect the systemic biological milieu involved in neointimal hyperplasia, thrombosis, and subsequent vascular access dysfunction. Although inflammation-based markers such as the neutrophil-to-lymphocyte ratio (NLR), platelet-to-lymphocyte ratio (PLR), and related indices have been associated with hemodialysis vascular access failure (3), the combined prognostic value of routine preoperative laboratory parameters and derived composite indices within an interpretable machine learning framework remains insufficiently clarified.

Against this background, this study aimed to develop and evaluate an interpretable machine learning model based on routine preoperative laboratory parameters and derived composite indices to predict AVF dysfunction within 1 year after AVF creation in patients with end-stage renal disease (ESRD).

## Methods

### Patients and variables

**Figure 1:**
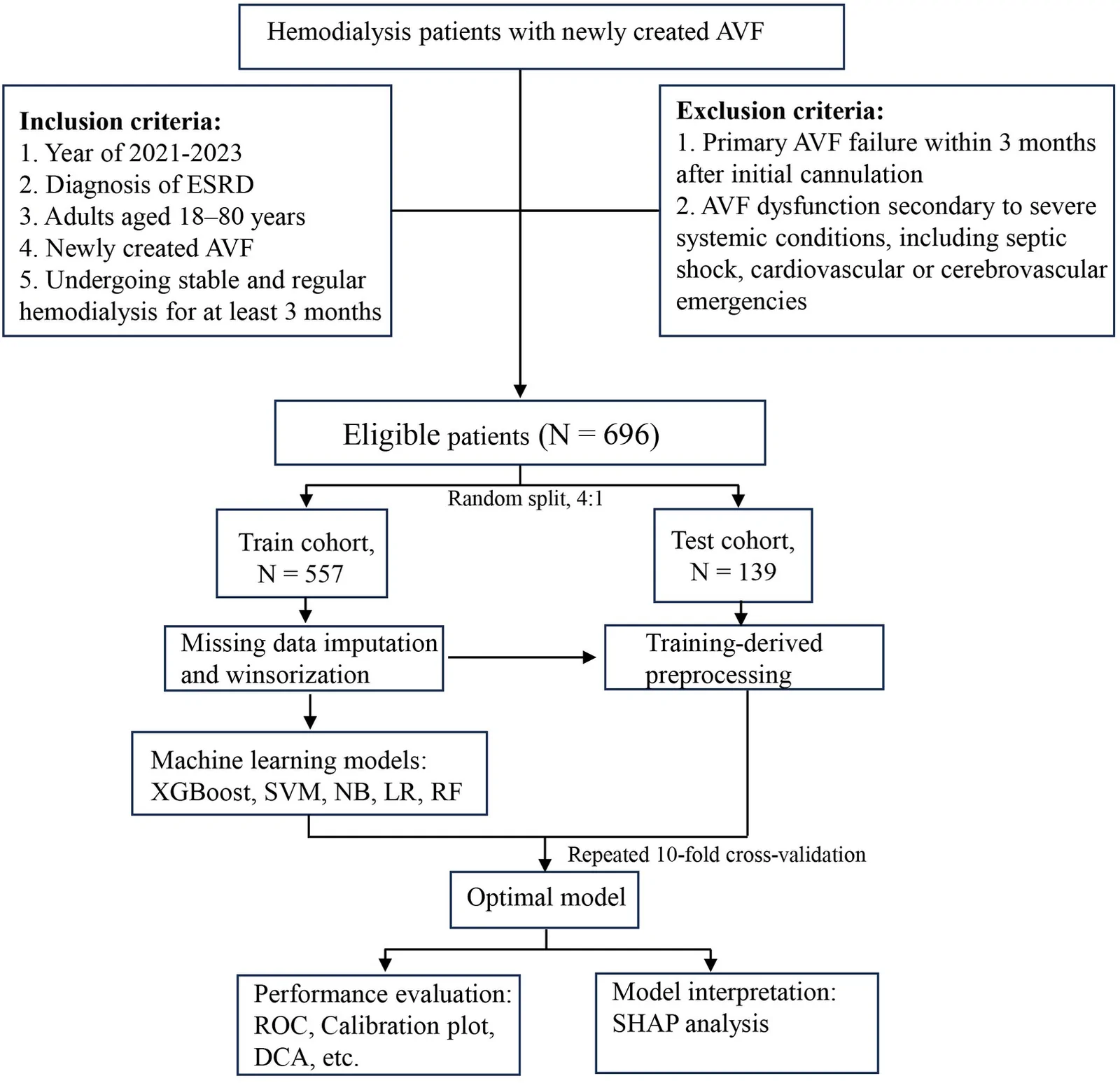
Study flow diagram. AVF, arteriovenous fistula; ESRD, end-stage renal disease; XGBoost, extreme gradient boosting; SVM, support vector machine; NB, Naive Bayes; LR, logistic regression; RF, random forest; ROC, receiver operating characteristic; DCA, decision curve analysis; SHAP, Shapley additive explanations.

This retrospective study included patients with ESRD who underwent creation of a new autogenous AVF for hemodialysis access at a tertiary hospital in Chongqing between January 2021 and December 2023. The inclusion and exclusion criteria, along with the study flow, are presented in Figure 1. The study was approved by the Ethics Committee of our hospital under the ethical approval number (B)KY2024178.

Baseline variables were abstracted from electronic medical records. Demographic characteristics included age and sex. Clinical features encompassed the cause of ESRD, the presence of diabetes mellitus, coronary heart disease, and dialysis vintage. Dialysis vintage was defined as the duration from initiation of maintenance hemodialysis to AVF creation and was recorded in months. Laboratory parameters were obtained from routine preoperative blood tests before AVF creation and included creatinine (Cr), absolute neutrophil count (ANC), absolute lymphocyte count (ALC), absolute monocyte count (AMC), platelet count (PLT), white blood cell count (WBC), alkaline phosphatase (ALP), hemoglobin (HB), glucose (GLU), albumin (ALB), C-reactive protein (CRP), corrected calcium (cCa), phosphate (Pi), and triglycerides (TG).

Based on these measurements, several composite indices were calculated, including albumin-to-alkaline phosphatase ratio (AAPR), C-reactive protein-to-albumin ratio (CAR), NLR, PLR, lymphocyte-to-monocyte ratio (LMR), platelet-to-neutrophil ratio (PNR), systemic immune-inflammation index (SII), systemic inflammation response index (SIRI), aggregate index of systemic inflammation (AISI), and the calcium–phosphorus product (CaP).

### Outcome

The primary outcome was the occurrence of AVF dysfunction within 1 year after AVF creation. Because patients with primary AVF failure within 3 months after surgery were excluded, the outcome represented late AVF dysfunction after initial AVF maturation rather than early maturation failure. For outcome assessment, patients were followed from AVF creation until the first AVF dysfunction event within 1 year or until completion of at least 12 months of event-free follow-up.

AVF dysfunction was defined as clinically relevant inadequate access function caused by AVF stenosis, occlusion, or thrombosis. Suspected dysfunction was identified by inadequate dialysis blood flow or difficulty in maintaining the prescribed extracorporeal blood flow during hemodialysis, together with abnormal clinical findings, such as weakened thrill or bruit, difficult cannulation, arm swelling, or loss of access flow. The diagnosis was further confirmed by Doppler ultrasound, angiographic findings, or endovascular/surgical intervention records when available. For patients who received maintenance hemodialysis outside our hospital after AVF creation, outcome status was determined using available follow-up records, intervention records, and telephone follow-up. Patients who developed AVF dysfunction within 1 year were classified into the dysfunction group, whereas those without evidence of dysfunction during the 1-year follow-up period were classified into the non-dysfunction group.

### Model construction, evaluation and visualization

The dataset was partitioned into the train cohort and test cohort in an approximately 4:1 ratio using stratified 5-fold partitioning based on AVF dysfunction status, with one fold designated as the test cohort and the remaining four folds combined as the train cohort, ensuring similar outcome distributions in both cohorts.

The train cohort underwent pre-processing to address missing data, outliers, and collinearity. Missing values in laboratory parameters were imputed using the median values estimated from the train cohort, and the same imputation values were then applied to the test cohort. Outliers were addressed through winsorization, with the 1st and 99th percentile thresholds estimated only from the train cohort and then applied to both cohorts. To reduce multicollinearity, highly correlated variables were identified in the train cohort and excluded from model construction. The same variable set was subsequently used in the test cohort.

Feature selection was performed within the train cohort using random forest (RF)-based recursive feature elimination (RFE). Candidate feature subset sizes of 3, 5, 6, 7, 8, 9, 10, 12, 15, and all available predictors were evaluated. The RFE process employed 5-fold cross-validation within the train cohort. The final feature subset was selected based on overall cross-validated performance, including the area under the receiver operating characteristic curve (AUC) and accuracy, while also considering model parsimony and clinical interpretability. When multiple feature subsets showed comparable performance, the smaller subset was preferred to reduce model complexity. The test cohort was not used during feature selection.

For model construction, five machine learning algorithms were trained using the train cohort, including extreme gradient boosting (XGBoost), RF, Naive Bayes (NB), support vector machine (SVM), and logistic regression (LR). Hyperparameter tuning was performed using random search, with repeated 10-fold cross-validation and three repeats within the train cohort. Candidate hyperparameter combinations were evaluated according to cross-validated AUC, and the optimal hyperparameters were selected for each model.

Model performance was evaluated in both the train cohort and test cohort. Discrimination was assessed using AUC and the area under the precision–recall curve (AUPRC). Classification performance was further evaluated using sensitivity, specificity, positive predictive value (PPV), negative predictive value (NPV), accuracy, and *F*1-score. The decision threshold for each model was determined in the train cohort using the Youden index and then applied unchanged to the test cohort for calculating threshold-based metrics. Calibration performance was assessed using Brier score, calibration intercept, and calibration slope in both cohorts. Calibration plots were generated for the final model to visually assess the agreement between predicted and observed probabilities. Decision curve analysis (DCA) was also performed for the final model to evaluate its potential clinical utility by estimating the net benefit across a range of threshold probabilities.

To enhance the interpretability of the final model, Shapley Additive Explanations (SHAP) values were used to assess the contribution of each feature to the model's predictions. SHAP visualizations were generated to provide insight into the importance of each variable in the final model. The contribution of each feature was quantified, allowing for a clear understanding of how each variable influenced the predicted outcomes.

### Statistical methods

Categorical variables were presented as counts and percentages. Continuous variables were expressed as median with interquartile range (IQR). Group comparisons for categorical variables were performed using the *χ*^2^ test or Fisher's exact test, as appropriate. Continuous variables were compared using the Mann–Whitney *U* test. Pairwise DeLong tests were used to compare the AUC of selected final model with the other machine learning models. The 95% confidence intervals (CIs) for AUC were estimated using the DeLong method. The 95% CIs for other model performance metrics, including AUPRC, sensitivity, specificity, PPV, NPV, accuracy, and *F*1-score, were estimated using bootstrap resampling with 1,000 iterations.

All tests were two-sided, and a *P* value <0.05 was considered statistically significant. All statistical analyses were performed using R software (version 4.4.0). Model development and evaluation were conducted using the caret, xgboost, randomForest, kernlab, naivebayes, pROC, PRROC, ggplot2, and shapviz packages (Supplementary Table 1). Random seeds were set before data partitioning, RFE, hyperparameter tuning, and bootstrap resampling to improve reproducibility.

## Results

A total of 696 patients with ESRD who underwent creation of a new AVF for hemodialysis between 2021 and 2023 were included. Within the 1-year outcome assessment after AVF creation, 130 patients (18.7%) developed AVF dysfunction. Compared with patients without AVF dysfunction, those with dysfunction were older (median age, 60 vs. 56 years; *P* = 0.008) and had a longer dialysis vintage (72.0 vs. 69.3 months, *P* = 0.014). The dysfunction group also showed a higher proportion of female patients (48.5% vs. 38.7%, *P* = 0.052) and diabetes mellitus (28.5% vs. 20.3%, *P* = 0.056), although these differences did not reach statistical significance. No significant differences were observed in the cause of ESRD or coronary heart disease between the two groups (Table 1).

**Table 1:** Comparison of baseline clinical characteristics between patients with and without AVF dysfunction.

Variables	All, *N* = 696 (%)	Non-dysfunctional AVF group, *N* = 566 (%)	Dysfunctional AVF group, *N* = 130 (%)	*P* value
Sex				0.052
Male	414 (59.5)	347 (61.3)	67 (51.5)	
Female	282 (40.5)	219 (38.7)	63 (48.5)	
Cause of ESRD				0.115
Glomerulonephritis	117 (16.8)	91 (16.1)	26 (20.0)	
Hypertension	37 (5.3)	31 (5.5)	6 (4.6)	
Diabetes	152 (21.8)	115 (20.3)	37 (28.5)	
Others	91 (13.1)	74 (13.1)	17 (13.1)	
Unknown	299 (43.0)	255 (45.0)	44 (33.8)	
Diabetes mellitus				0.056
Yes	152 (21.8)	115 (20.3)	37 (28.5)	
No	544 (78.2)	451 (79.7)	93 (71.5)	
Coronary heart disease				0.859
Yes	37 (5.3)	31 (5.5)	6 (4.6)	
No	659 (94.7)	535 (94.5)	124 (95.4)	
Age, years	57 (43, 68)	56 (42, 68)	60 (48, 70)	0.008
Dialysis vintage, months	69.3 (32.0, 93.0)	69.3 (32.0, 81.5)	72.0 (33.3, 108.0)	0.014

Data are presented as *n* (%) for categorical variables and median (interquartile range) for continuous variables. AVF, arteriovenous fistula; ESRD, end-stage renal disease.

Using stratified partitioning at an approximately 4:1 ratio, the cohort was divided into the train cohort (*n* = 557) and test cohort (*n* = 139). Baseline clinical characteristics and laboratory parameters were well balanced between the two cohorts, with no statistically significant differences observed (Supplementary Table 2). The proportions of missing laboratory values were generally low, ranging from 0% to 7.47% in the overall cohort. CRP had the highest missing proportion, followed by GLU, WBC, ALB, Cr, PLT, ANC, and ALP (Supplementary Table 3). Missing laboratory values were imputed using median values estimated from the train cohort, and the same imputation values were applied to the test cohort. After imputation, derived composite indices were calculated for subsequent feature selection and model construction.

**Figure 2:**
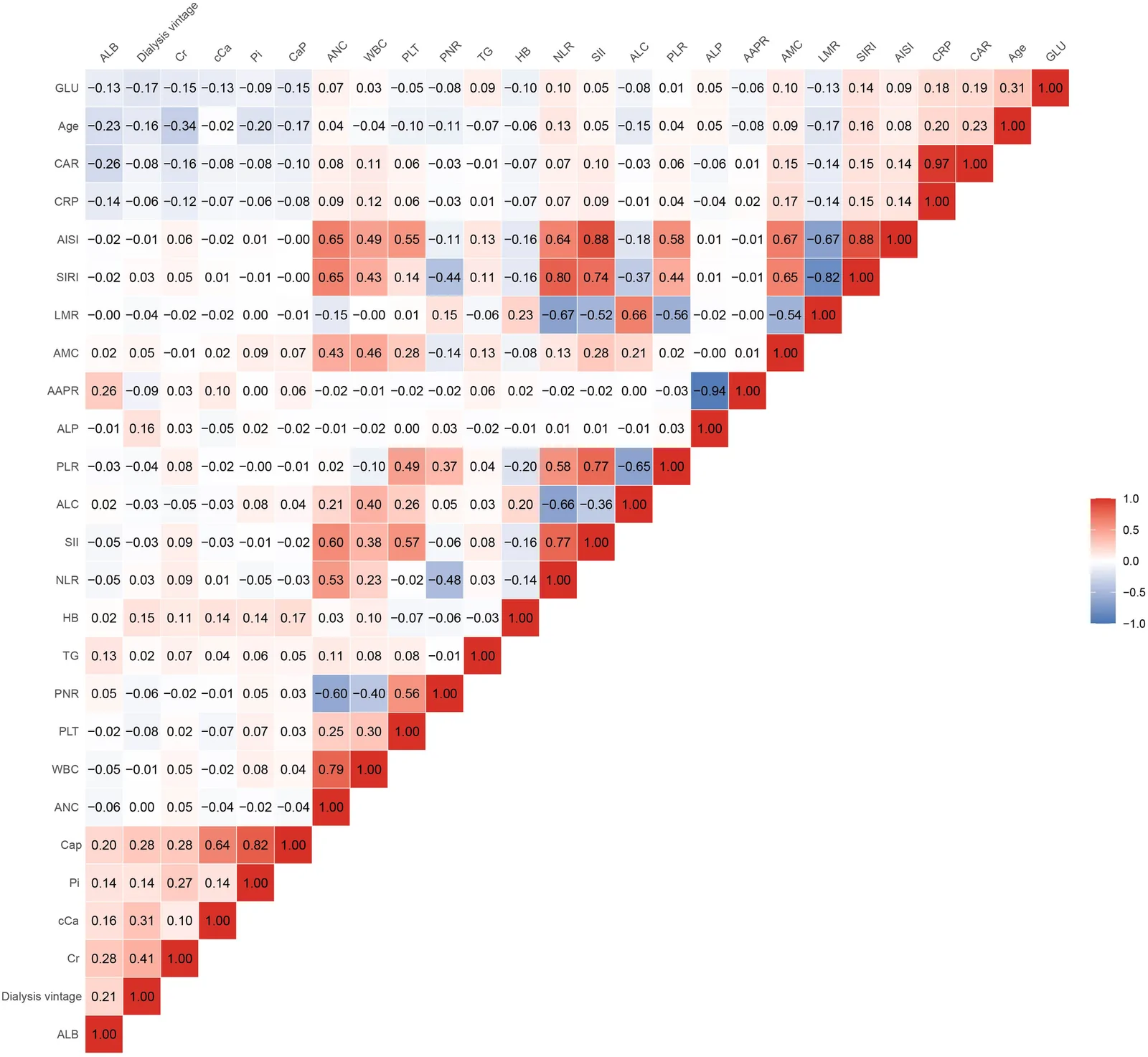
Correlation heatmap of continuous clinical and laboratory variables in the train cohort. The heatmap shows pairwise Spearman correlation coefficients among baseline variables and derived composite indices. ALB, albumin; Cr, creatinine; cCa, corrected calcium; Pi, phosphate; CaP, calcium–phosphorus product; ANC, absolute neutrophil count; WBC, white blood cell count; PLT, platelet count; PNR, platelet-to-neutrophil ratio; TG, triglycerides; HB, hemoglobin; NLR, neutrophil-to-lymphocyte ratio; SII, systemic immune-inflammation index; ALC, absolute lymphocyte count; PLR, platelet-to-lymphocyte ratio; ALP, alkaline phosphatase; AAPR, albumin-to-alkaline phosphatase ratio; AMC, absolute monocyte count; LMR, lymphocyte-to-monocyte ratio; SIRI, systemic inflammation response index; AISI, aggregate index of systemic inflammation; CRP, C-reactive protein; CAR, C-reactive protein-to-albumin ratio; GLU, glucose.

Pairwise Spearman correlations among continuous variables and derived indices were calculated in the train cohort (Figure 2). Several variable pairs showed strong correlations with an absolute correlation coefficient greater than 0.70, including ANC with WBC (*r* = 0.79), ALP with AAPR (*r* = −0.94), CRP with CAR (*r* = 0.97), NLR with SII and SIRI (*r* = 0.77 and 0.80, respectively), PLR with SII (*r* = 0.77), LMR with SIRI (*r* = −0.82), SII with SIRI and AISI (*r* = 0.74 and 0.88, respectively), SIRI with AISI (*r* = 0.88), and Pi with CaP (*r* = 0.82). Accordingly, WBC, AAPR, CRP, SII, SIRI, Pi, and cCa, were excluded from subsequent model construction to reduce multicollinearity and avoid redundancy between original laboratory variables and formula-derived composite indices.

**Figure 3:**
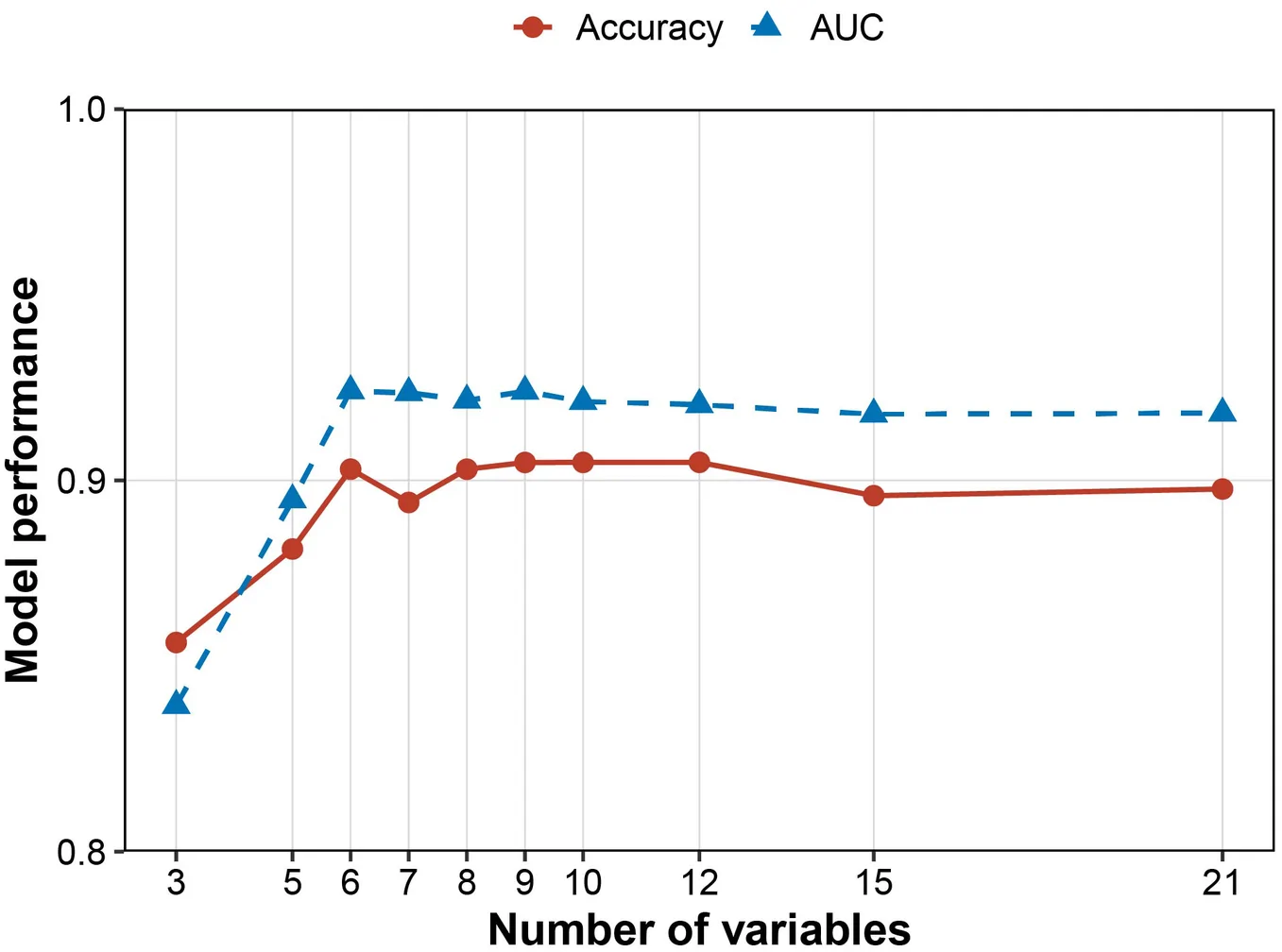
Feature subset selection based on recursive feature elimination. Model performance was evaluated across different numbers of candidate variables using cross-validation in the train cohort. Accuracy and AUC increased when the number of variables increased from 3 to 6 and then remained relatively stable with additional variables. AUC, area under the receiver operating characteristic curve.

RF-based RFE analysis was performed in the train cohort to determine the optimal feature subset for model construction. AUC increased markedly when the number of predictors increased from 3 to 6 and then remained relatively stable with additional predictors (Figure 3). Accuracy showed a similar pattern, with only minimal changes after six predictors were included. Because larger feature subsets did not provide substantial additional improvement, a six-variable subset was selected to balance predictive performance and model parsimony. The final predictors were HB, AISI, dialysis vintage, CaP, TG, and CAR.

**Figure 4:**
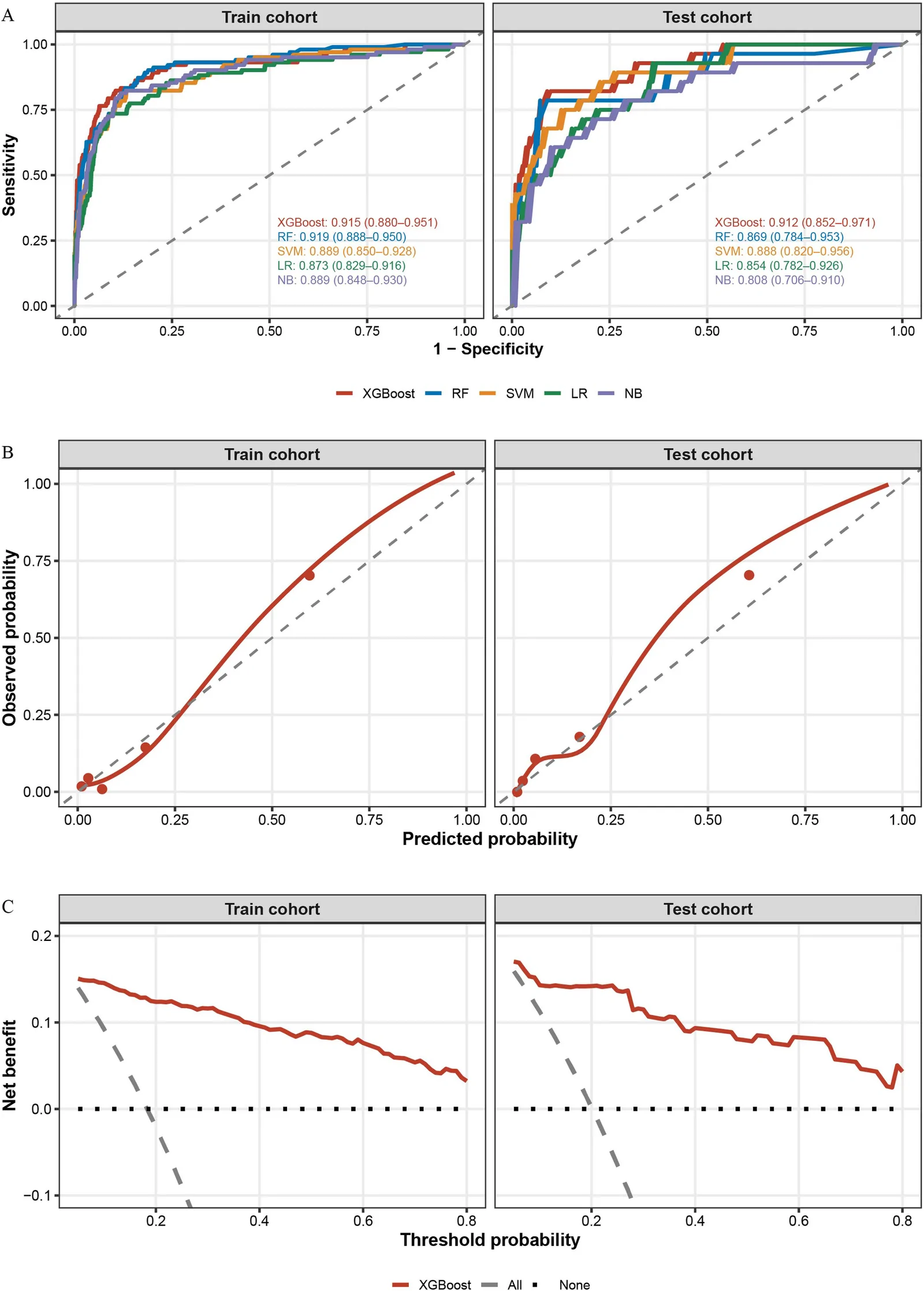
Model performance of the five machine learning models in the train and test cohorts. **(A)** Receiver operating characteristic curves of XGBoost, RF, SVM, LR, and NB models in the train and test cohorts. The AUC values with 95% confidence intervals are shown in each panel. **(B)** Calibration plots of the final XGBoost model in the train and test cohorts. **(C)** Decision curve analysis of the final XGBoost model in the train and test cohorts. XGBoost, extreme gradient boosting; RF, random forest; SVM, support vector machine; LR, logistic regression; NB, Naive Bayes; AUC, area under the receiver operating characteristic curve.

Five machine learning models were constructed using the six selected predictors. In the train cohort, RF achieved the highest AUC (0.919, 95% CI 0.888–0.950), followed closely by XGBoost (0.915, 95% CI 0.880–0.951). In the test cohort, XGBoost showed the highest AUC (0.912, 95% CI 0.852–0.971), followed by SVM (0.888, 95% CI 0.820–0.956), RF (0.869, 95% CI 0.784–0.953), LR (0.854, 95% CI 0.782–0.926), and NB (0.808, 95% CI 0.706–0.910) (Figure 4A). Pairwise DeLong tests showed similar patterns in both cohorts. XGBoost had significantly higher AUCs than LR and NB, whereas the AUC differences between XGBoost and RF or SVM did not reach statistical significance in both cohorts (Supplementary Table 4). Beyond AUC, XGBoost demonstrated favorable overall performance in both cohorts. In the train cohort, XGBoost achieved the highest AUPRC, PPV, NPV, accuracy, and *F*1-score, while maintaining balanced sensitivity and specificity. In the test cohort, XGBoost showed the most favorable overall performance, with the highest AUPRC, sensitivity, specificity, PPV, NPV, accuracy, and *F*1-score among the five models (Table 2). Accordingly, XGBoost was selected as the final model based on its overall performance. For the final XGBoost model, the tuned hyperparameters included the number of boosting rounds, maximum tree depth, learning rate, minimum loss reduction, column subsampling, minimum child weight, and row subsampling. Early stopping was not used. The optimal hyperparameters of the final XGBoost model are provided in Supplementary Table 5.

**Table 2:** Performance of five machine learning models for 1-year arteriovenous fistula dysfunction in the train and test cohorts.

Cohorts	ML algorithms	AUPRC	Sensitivity	Specificity	PPV	NPV	Accuracy	*F*1_score
Train cohort	XGBoost	0.805 (0.732–0.866)	0.824 (0.746–0.892)	0.895 (0.864–0.923)	0.636 (0.559–0.715)	0.958 (0.938–0.976)	0.882 (0.855–0.908)	0.718 (0.654–0.780)
	RF	0.780 (0.702–0.848)	0.902 (0.842–0.959)	0.811 (0.774–0.847)	0.517 (0.445–0.588)	0.974 (0.957–0.989)	0.828 (0.795–0.858)	0.657 (0.589–0.716)
	NB	0.726 (0.634–0.808)	0.814 (0.726–0.888)	0.881 (0.850–0.909)	0.606 (0.525–0.681)	0.955 (0.933–0.974)	0.869 (0.840–0.896)	0.695 (0.621–0.754)
	SVM	0.737 (0.649–0.810)	0.824 (0.750–0.898)	0.868 (0.835–0.898)	0.583 (0.508–0.662)	0.956 (0.937–0.975)	0.860 (0.831–0.887)	0.683 (0.611–0.742)
	LR	0.706 (0.615–0.778)	0.735 (0.645–0.817)	0.908 (0.881–0.933)	0.641 (0.550–0.726)	0.939 (0.914–0.959)	0.876 (0.849–0.901)	0.685 (0.604–0.752)
Test cohort	XGBoost	0.787 (0.630–0.902)	0.821 (0.667–0.958)	0.901 (0.842–0.955)	0.676 (0.516–0.821)	0.952 (0.907–0.990)	0.885 (0.834–0.935)	0.742 (0.600–0.850)
	RF	0.738 (0.569–0.862)	0.786 (0.625–0.926)	0.811 (0.737–0.882)	0.512 (0.368–0.652)	0.938 (0.885–0.980)	0.806 (0.741–0.871)	0.620 (0.476–0.737)
	NB	0.566 (0.373–0.774)	0.679 (0.500–0.850)	0.811 (0.738–0.881)	0.475 (0.324–0.633)	0.909 (0.853–0.961)	0.784 (0.719–0.849)	0.559 (0.407–0.686)
	SVM	0.750 (0.601–0.873)	0.750 (0.576–0.906)	0.838 (0.770–0.901)	0.538 (0.371–0.683)	0.930 (0.875–0.979)	0.820 (0.755–0.878)	0.627 (0.481–0.753)
	LR	0.653 (0.474–0.810)	0.571 (0.385–0.760)	0.874 (0.812–0.931)	0.533 (0.364–0.720)	0.890 (0.830–0.946)	0.813 (0.748–0.878)	0.552 (0.383–0.700)

ML, machine learning; AUPRC, area under the precision-recall curve; PPV, positive predictive value; NPV, negative predictive value; XGBoost, extreme gradient boosting; RF, random forest; NB, naive Bayes; SVM, support vector machine; LR, logistic regression.

The XGBoost model showed acceptable calibration in both cohorts. Among the five models, XGBoost had the lowest Brier score in both the train cohort (0.074) and the test cohort (0.086) (Supplementary Table 6). The calibration intercepts were 0.109 in the train cohort and 0.366 in the test cohort, and the calibration slopes were 1.261 and 1.143, respectively. These results suggested generally acceptable probabilistic calibration, although the positive calibration intercept in the test cohort indicated a tendency toward mild underestimation of the observed risk. Calibration plots also demonstrated generally good agreement between predicted and observed probabilities (Figure 4B). Decision curve analysis showed that the XGBoost-based strategy provided a positive net benefit compared with the standard all and none reference strategies across a range of threshold probabilities (Figure 4C).

**Figure 5:**
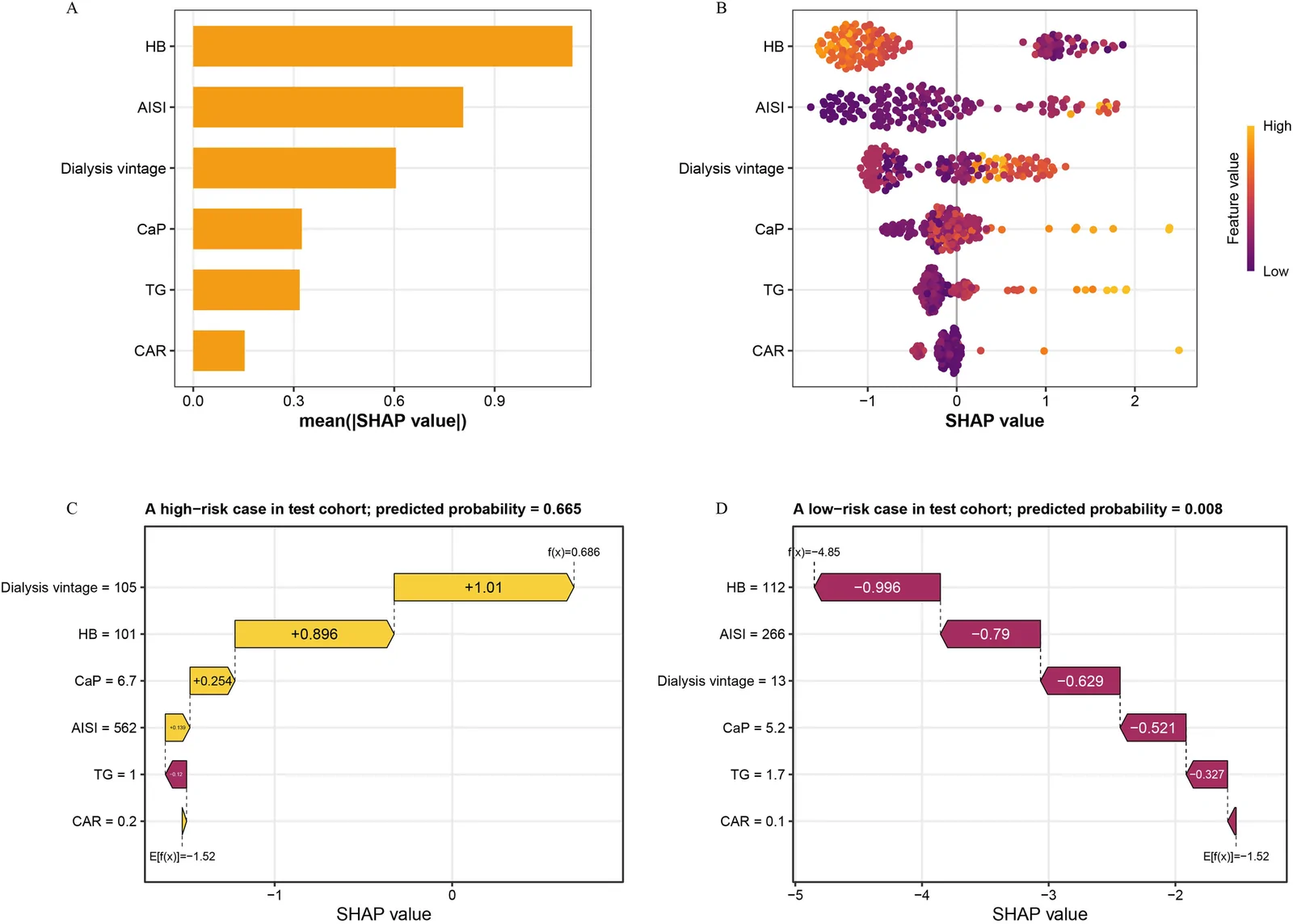
SHAP-based interpretation of the XGBoost model. **(A)** SHAP feature importance plot showing the mean absolute SHAP value of each predictor. **(B)** SHAP summary plot showing the distribution and direction of feature contributions to model predictions. **(C)** SHAP waterfall plot for a representative high-risk case in the test cohort. **(D)** SHAP waterfall plot for a representative low-risk case in the test cohort. SHAP, Shapley Additive Explanations; XGBoost, extreme gradient boosting; HB, hemoglobin; AISI, aggregate index of systemic inflammation; CaP, calcium–phosphorus product; TG, triglycerides; CAR, C-reactive protein-to-albumin ratio.

SHAP analysis was performed to interpret the final XGBoost model and quantify the contribution of each predictor to the predicted risk of AVF dysfunction within one year. Among the six predictors included in the model, HB had the largest mean absolute SHAP value, followed by AISI, dialysis vintage, CaP, TG, and CAR (Figure 5A). The SHAP summary plot showed that lower HB values were generally associated with positive SHAP values, corresponding to an increased predicted risk, whereas higher HB values tended to reduce the predicted risk. Higher AISI, longer dialysis vintage, higher CaP, and higher TG were generally associated with increased predicted risk. CAR contributed less to the overall model prediction than the other predictors, but higher CAR values tended to shift the prediction toward increased AVF dysfunction risk in a subset of patients (Figure 5B). Patient-level SHAP waterfall plots further illustrated how individual feature values contributed to model predictions. In a representative high-risk patient, longer dialysis vintage, lower HB, higher CaP, and higher AISI shifted the model output toward a higher predicted probability of AVF dysfunction. In contrast, in a representative low-risk patient, higher HB, lower AISI, shorter dialysis vintage, and lower CaP shifted the prediction toward a lower risk estimate (Figures 5C,D).

## Discussion

In this study, we developed and evaluated an interpretable machine learning model to predict AVF dysfunction within 1 year after AVF creation in patients with ESRD using routinely available preoperative clinical and laboratory variables. The final XGBoost model incorporating six predictors—HB, AISI, dialysis vintage, CaP, TG, and CAR—showed favorable discrimination and acceptable calibration in both the train and test cohorts. SHAP analysis provided additional interpretability by explaining the contribution of individual predictors to model-derived risk estimates. In clinical practice, the XGBoost-derived predicted probability should be interpreted as a continuous estimate of 1-year dysfunction risk, which may help identify patients who require closer attention after initial AVF maturation. Because the model was developed from preoperative clinical and laboratory variables, its potential value lies mainly in supporting postoperative follow-up planning and surveillance prioritization. Patients with predicted probabilities above the overall event rate observed in this cohort, 18.7%, may be considered to have above-average risk and may warrant closer postoperative follow-up, closer monitoring of dialysis blood flow and physical signs of access dysfunction, and earlier Doppler ultrasound evaluation when clinically indicated. However, this value should be regarded as an exploratory reference rather than a fixed clinical cutoff, and external validation is needed before clinical implementation.

Previous machine learning studies have addressed different AVF-related outcomes, but their clinical targets and input variables vary substantially. Peralta et al. developed an XGBoost-based model to predict AVF failure within 3 months among in-centre hemodialysis patients using dialysis clinic data (6). Heindel et al. proposed the PREDICT-AVF tool to predict successful unassisted use of newly created radiocephalic AVFs, incorporating clinical and ultrasound-based information (7). Li et al. developed models for 1-year successful clinical use of arteriovenous access using vascular registry data (8), whereas Fitzgibbon et al. extended this framework to long-term radiocephalic AVF patency prediction using prospective trial data and postoperative ultrasound measurements (10). Other studies focused on specific failure-related events, including AVF stenosis, thrombosis, and postoperative occlusion (9, 11, 12). In addition, Wang et al. constructed a conventional clinical prediction model for AVF dysfunction that included clinical, laboratory, and anatomical variables such as anastomotic diameter (13). Compared with these studies, our model focused on clinically relevant AVF dysfunction within 1 year after AVF creation among patients who had achieved initial maturation. The model was based on routinely available preoperative clinical and laboratory variables, rather than postoperative dialysis information, vascular sound signals, ultrasound-derived anatomical features, or surgical variables. Therefore, our model is not intended to replace anatomical or procedure-based prediction models for fistula planning. Instead, it may provide complementary value for early risk estimation and postoperative surveillance prioritization when detailed anatomical or postoperative monitoring data are unavailable.

In the final XGBoost model, HB, AISI, and dialysis vintage were the leading contributors to model predictions according to the SHAP importance ranking. Lower HB values were generally associated with higher model-predicted risk, whereas higher HB values tended to shift predictions toward lower risk. This finding is clinically plausible, as severe anemia has been linked to poorer vascular access survival, while correction of HB within the recommended range does not appear to increase the risk of access thrombosis (15, 16). Dialysis vintage was also among the most influential predictors. Longer dialysis vintage and greater cumulative dialysis exposure have been associated with adverse AVF outcomes in previous studies, including lower maturation rates and fistula failure, likely reflecting the impact of chronic uremia, repeated cannulation, and dialysis-related hemodynamic stress on the vascular wall (15, 16). Inflammation-related indicators also contributed to the final model. Higher AISI values were generally associated with higher model-predicted risk of AVF dysfunction. AISI has been reported as a predictor of venous thrombosis (17). As a composite index incorporating neutrophils, monocytes, platelets, and lymphocytes, AISI may reflect a systemic pro-inflammatory, pro-thrombotic milieu that promotes neointimal hyperplasia, endothelial dysfunction, and thrombosis within the access circuit (18, 19). CAR, which combines CRP and albumin, can be regarded as an integrative marker of inflammation and nutritional status and is closely linked to the malnutrition–inflammation–atherosclerosis complex. Higher CAR has been associated with increased mortality and cardiovascular events in dialysis and kidney disease populations (20, 21). Although CAR showed a relatively smaller overall SHAP contribution than the leading predictors, its inclusion in the final model suggests that inflammatory–nutritional status may provide complementary predictive information for AVF dysfunction.

Both TG and the CaP were associated with higher model-predicted risk of AVF dysfunction. Previous studies have suggested that dyslipidemia and higher cholesterol burden are related to impaired fistula patency and thrombosis, supporting a potential role of metabolic and atherosclerotic stress in access failure (22). Consistently, TG and TG-based composite lipid indices have been associated with reduced AVF patency and an increased risk of access failure (23, 24). These findings are consistent with the possibility that adverse lipid profiles may contribute to intimal hyperplasia, endothelial injury, and thrombotic events within the access circuit. The role of CaP in our model is also biologically plausible. Elevated Pi and CaP are established risk factors for vascular calcification and cardiovascular mortality in hemodialysis patients (25, 26), and higher CaP levels have been associated with increased AVF failure and re-operation, presumably through increased arterial stiffness and impaired vascular remodeling (27).

Compared with conventional regression-based risk scores for AVF outcomes, the use of an XGBoost model in this study allowed us to flexibly capture potential non-linear effects and interactions among clinical, inflammatory, and metabolic variables without imposing strong parametric assumptions. By restricting the input to routinely available preoperative clinical and laboratory indices, the model remains pragmatically applicable in clinical practice while still achieving good discriminatory performance, which is consistent with previous reports applying tree-based ensemble methods such as XGBoost to predict AVF stenosis or failure in hemodialysis populations (9). In addition, the use of SHAP values to interrogate the XGBoost model provides case-level interpretability and clarifies how specific features—such as dialysis vintage, TG, CAR, AISI, and CaP—shape the predicted risk, thereby enhancing the transparency and clinical interpretability of the model.

AVF dysfunction is influenced by anatomical, surgical, and dialysis-care factors in addition to systemic clinical and laboratory variables. Recent evidence indicates that smaller arterial diameter, smaller venous diameter, and distal fistula location are associated with early AVF failure or non-maturation (28). Ultrasound-derived vascular parameters are also important. Postoperative venous diameter has been shown to predict early AVF failure (29), and anastomotic diameter, venous diameter, and access flow have been associated with AVF maturation (30). Surgical configuration may further affect local hemodynamics and access outcomes, as anastomotic angle has been suggested to influence AVF maturation, patency, and reintervention risk (31). In addition, cannulation technique may also affect AVF survival (32). These findings indicate that anatomical and procedural variables provide clinically relevant information that was not captured in the present laboratory-based model. Therefore, although our model may provide a pragmatic reference for postoperative surveillance based on preoperative clinical and laboratory variables, the absence of these variables may limit its predictive completeness, transportability, and clinical use. The current model may therefore be better interpreted as an initial risk-assessment reference rather than a fully comprehensive model incorporating anatomical, procedural, and laboratory information. Future models integrating laboratory variables with standardized vascular ultrasound measurements, surgical details, and anastomotic characteristics may improve predictive performance and provide a more complete basis for clinical assessment.

This study has several limitations. First, it was a retrospective, single-center analysis, which may limit generalizability and introduce selection bias or residual confounding. Although an internal train–test split was performed, external validation in independent cohorts is still required to assess model transportability and calibration. Second, the model was built only on baseline preoperative clinical and laboratory variables. Important anatomical and procedural factors were not included, which may limit predictive completeness and clinical applicability.

## Conclusion

An interpretable XGBoost model based on six preoperative variables—HB, AISI, dialysis vintage, CaP, TG, and CAR—was developed to predict AVF dysfunction within 1 year after AVF creation in patients with ESRD. The model showed favorable performance and may help identify patients at higher risk of late AVF dysfunction. For these high-risk patients, closer postoperative surveillance may be considered. Further external validation is required before clinical application.

## Data Availability

The raw data supporting the conclusions of this article will be made available by the authors, without undue reservation.
