# Supplementary material for: An interpretable XGBoost model for predicting arteriovenous fistula dysfunction in end stage renal disease
Source: Front Surg. 2026 Aug 17;13:1860573. doi: 10.3389/fsurg.2026.1860573 (PMC13526567; doi:10.3389/fsurg.2026.1860573)
Supplement: Supplementary file 1 [file Table1.docx]

Supplementary Table 1: Software packages and versions for model development and evaluation.

| **Purpose** | **Software / package** | **Version** |
| --- | --- | --- |
| Statistical environment | R | 4.4.0 |
| Descriptive statistics and group comparisons | tableone | 0.13.2 |
| Data preprocessing and manipulation | dplyr; tidyr; tibble | 1.1.4; 1.3.1; 3.3.0 |
| Model training, resampling, RFE, and model performance metrics | caret | 7.0-1 |
| XGBoost model | xgboost | 1.7.11.1 |
| Random forest model | randomForest | 4.7-1.2 |
| Support vector machine | kernlab | 0.9-33 |
| Naive Bayes model | naivebayes | 1.0.0 |
| ROC analysis and DeLong test | pROC | 1.19.0.1 |
| Precision–recall analysis | PRROC | 1.4 |
| SHAP interpretation | shapviz | 0.10.3 |
| Visualization | ggplot2; patchwork; reshape2 | 4.0.1; 1.3.2; 1.4.5 |

RFE, recursive feature elimination; XGBoost, extreme gradient boosting; ROC, receiver operating characteristic; AUC, area under the receiver operating characteristic curve; CI, confidence interval; PRROC, precision–recall receiver operating characteristic; SHAP, Shapley additive explanations.

Supplementary Table 2: Comparison of baseline clinical characteristics and laboratory parameters between train cohort and test cohort.

| **Variables** | Train cohort,  N = 557 | Test cohort,  N = 139 | P value |
| --- | --- | --- | --- |
| **Sex** |  |  | 0.820 |
| Male | 224 (40.2) | 58 (41.7) |  |
| Female | 333 (59.8) | 81 (58.3) |  |
| **Cause of ESRD** |  |  | 0.857 |
| Glomerulonephritis | 98 (17.6) | 19 (13.7) |  |
| Hypertension | 29 (5.2) | 8 (5.8) |  |
| Diabetes | 121 (21.7) | 31 (22.3) |  |
| Others | 73 (13.1) | 18 (12.9) |  |
| Unknown | 236 (42.4) | 63 (45.3) |  |
| **Diabetes mellitus** |  |  | 0.974 |
| Yes | 121 (21.7) | 31 (22.3) |  |
| No | 436 (78.3) | 108 (77.7) |  |
| **Coronary heart disease** |  |  | 0.963 |
| Yes | 29 (5.2) | 8 (5.8) |  |
| No | 528 (94.8) | 131 (94.2) |  |
| **Smoking history** |  |  | 0.820 |
| Yes | 333 (59.8) | 81 (58.3) |  |
| No | 224 (40.2) | 58 (41.7) |  |
| **Age, years** | 56 (44, 68) | 62 (43, 70) | 0.145 |
| **Dialysis vintage, months** | 69.3 (32.0, 86.0) | 69.3 (35.5, 103.0) | 0.621 |
| **Laboratory parameters** |  |  |  |
| Cr (µmol/L) | 832.0 (569.9, 1077.9); n=543 | 881.6 (593.1, 1066.5); n=135 | 0.427 |
| ANC (×10⁹/L) | 4.34 (3.44, 5.74); n=548 | 4.49 (3.77, 5.71); n=137 | 0.424 |
| ALC (×10⁹/L) | 1.18 (0.92, 1.60); n=554 | 1.20 (0.92, 1.56); n=139 | 0.737 |
| AMC (×10⁹/L) | 0.47 (0.36, 0.60); n=552 | 0.47 (0.36, 0.62); n=138 | 0.735 |
| PLT (×10⁹/L) | 180 (136, 227); n=543 | 178 (146, 223); n=136 | 0.767 |
| WBC (×10⁹/L) | 6.44 (5.11, 8.05); n=536 | 6.72 (5.59, 7.97); n=137 | 0.244 |
| ALP (U/L) | 89.0 (67.0, 118.8); n=550 | 86.0 (66.5, 110.5); n=135 | 0.408 |
| HB (g/L) | 110 (95, 118); n=557 | 108 (95, 116); n=139 | 0.339 |
| GLU (mmol/L) | 6.0 (5.0, 9.0); n=530 | 6.0 (5.0, 9.0); n=133 | 0.900 |
| ALB (g/L) | 40.0 (37.0, 42.0); n=539 | 39.0 (37.0, 42.0); n=136 | 0.731 |
| CRP (mg/L) | 6.0 (2.0, 17.0); n=517 | 8.0 (2.0, 16.5); n=127 | 0.300 |
| cCa (mmol/L) | 2.6 (2.3, 2.9); n=557 | 2.5 (2.3, 3.0); n=139 | 0.878 |
| Pi (mmol/L) | 2.4 (2.1, 2.8); n=557 | 2.5 (2.2, 2.8); n=139 | 0.360 |
| TG (mmol/L) | 2.0 (1.6, 2.6); n=557 | 2.0 (1.7, 2.6); n=139 | 0.932 |

Data are presented as n (%) for categorical variables and median (interquartile range) for continuous variables. ESRD, end-stage renal disease; Cr, creatinine; ANC, absolute neutrophil count; ALC, absolute lymphocyte count; AMC, absolute monocyte count; PLT, platelet count; WBC, white blood cell count; ALP, alkaline phosphatase; HB, hemoglobin; GLU, glucose; ALB, albumin; CRP, C-reactive protein; cCa, corrected calcium; Pi, serum phosphorus; TG, triglycerides.

Supplementary Table 3: Missing data proportions of laboratory parameters in the overall, train, and test cohorts

| **Laboratory parameters** | All, % | Train cohort, % | Test cohort, % |
| --- | --- | --- | --- |
| Cr (µmol/L) | 2.59 | 2.51 | 2.88 |
| ANC (×10⁹/L) | 1.58 | 1.62 | 1.44 |
| ALC (×10⁹/L) | 0.43 | 0.54 | 0 |
| AMC (×10⁹/L) | 0.86 | 0.90 | 0.72 |
| PLT (×10⁹/L) | 2.44 | 2.51 | 2.16 |
| WBC (×10⁹/L) | 3.30 | 3.77 | 1.44 |
| ALP (U/L) | 1.58 | 1.26 | 2.88 |
| HB (g/L) | 0 | 0 | 0 |
| GLU (mmol/L) | 4.74 | 4.85 | 4.32 |
| ALB (g/L) | 3.02 | 3.23 | 2.16 |
| CRP (mg/L) | 7.47 | 7.18 | 8.63 |
| cCa (mmol/L) | 0 | 0 | 0 |
| Pi (mmol/L) | 0 | 0 | 0 |
| TG (mmol/L) | 0 | 0 | 0 |

Data are presented as percentages of missing values. Percentages were calculated as the number of missing observations divided by

the total number of patients in the corresponding cohort: overall cohort, N = 696; train cohort, N = 557; test cohort, N = 139.

Supplementary Table 4: Pairwise DeLong tests comparing the AUC of XGBoost with other machine learning models.

| Cohort | Comparison | AUC of XGBoost | AUC of comparator | AUC difference | P value |
| --- | --- | --- | --- | --- | --- |
| Train cohort | XGBoost vs. RF | 0.915 (0.880-0.951) | 0.919 (0.888-0.950) | -0.003 | 0.747 |
|  | XGBoost vs. NB | 0.915 (0.880-0.951) | 0.889 (0.848-0.930) | 0.026 | 0.013 |
|  | XGBoost vs. SVM | 0.915 (0.880-0.951) | 0.889 (0.850-0.928) | 0.026 | 0.099 |
|  | XGBoost vs. LR | 0.915 (0.880-0.951) | 0.873 (0.829-0.916) | 0.043 | < 0.001 |
| Test  cohort | XGBoost vs. RF | 0.912 (0.852-0.971) | 0.869 (0.784-0.953) | 0.043 | 0.098 |
|  | XGBoost vs. NB | 0.912 (0.852-0.971) | 0.808 (0.706-0.910) | 0.104 | 0.038 |
|  | XGBoost vs. SVM | 0.912 (0.852-0.971) | 0.888 (0.820-0.956) | 0.024 | 0.224 |
|  | XGBoost vs. LR | 0.912 (0.852-0.971) | 0.854 (0.782-0.926) | 0.058 | 0.029 |

XGBoost, extreme gradient boosting; RF, random forest; NB, naive Bayes; SVM, support vector machine; LR, logistic regression; AUC, area under the receiver operating characteristic curve.

Supplementary Table 5: Optimal hyperparameters of the final XGBoost model.

| Number of boosting rounds, nrounds | 702 |
| --- | --- |
| Maximum tree depth, max_depth | 7 |
| Learning rate, eta | 0.0419 |
| Minimum loss reduction, gamma | 2.8147 |
| Column subsampling, colsample_bytree | 0.4463 |
| Minimum child weight, min_child_weight | 1 |
| Row subsampling, subsample | 0.6848 |
| L1 regularization, alpha | Default setting; not separately tuned |
| L2 regularization, lambda | Default setting; not separately tuned |
| Early stopping | Not used |

Supplementary Table 6: Calibration performance of five machine learning models in the train and test cohorts

| Cohort | Model | Brier score | Calibration intercept | Calibration slope |
| --- | --- | --- | --- | --- |
| Train cohort | XGBoost | 0.074 | 0.109 | 1.261 |
|  | NB | 0.085 | 0.264 | 0.841 |
|  | RF | 0.077 | -0.034 | 0.933 |
|  | SVM | 0.087 | -0.040 | 1.158 |
|  | LR | 0.091 | 0.003 | 0.932 |
| Test cohort | XGBoost | 0.086 | 0.366 | 1.143 |
|  | NB | 0.120 | 0.511 | 0.445 |
|  | RF | 0.094 | 0.260 | 0.545 |
|  | SVM | 0.093 | 0.065 | 1.202 |
|  | LR | 0.113 | 0.038 | 0.842 |

XGBoost, extreme gradient boosting; RF, random forest; NB, naive Bayes; SVM, support vector machine; LR, logistic regression.
